# Hypothermia in a surgical intensive care unit

**DOI:** 10.1186/1471-2253-5-7

**Published:** 2005-06-06

**Authors:** Fernando J Abelha, Maria A Castro, Aida M Neves, Nuno M Landeiro, Cristina C Santos

**Affiliations:** 1 Department of Anesthesia and Intensive Care, Hospital de São João, Porto, Portugal; 2 Biostatistics and Medical Informatics Department, Faculty of Medicine at the University of Porto, Portugal

## Abstract

**Background:**

Inadvertent hypothermia is not uncommon in the immediate postoperative period and it is associated with impairment and abnormalities in various organs and systems that can lead to adverse outcomes. The aim of this study was to estimate the prevalence, the predictive factors and outcome of core hypothermia on admission to a surgical ICU.

**Methods:**

All consecutive 185 adult patients who underwent scheduled or emergency noncardiac surgery admitted to a surgical ICU between April and July 2004 were admitted to the study. Tympanic membrane core temperature (Tc) was measured before surgery, on arrival at ICU and every two hours until 6 hours after admission. The following variables were also recorded: age, sex, body weight and height, ASA physical status, type of surgery, magnitude of surgical procedure, anesthesia technique, amount of intravenous fluids administered during anesthesia, use of temperature monitoring and warming techniques, duration of the anesthesia, ICU length of stay, hospital length of stay and SAPS II score. Patients were classified as either hypothermic (Tc ≤ 35°C) or normothermic (Tc> 35°C). Univariate analysis and multiple regression binary logistic with an odds ratio (OR) and its 95% Confidence Interval (95%CI) were used to compare the two groups of patients and assess the relationship between each clinical predictor and hypothermia. Outcome measured as ICU length of stay and mortality was also assessed.

**Results:**

Prevalence of hypothermia on ICU admission was 57.8%. In univariate analysis temperature monitoring, use of warming techniques and higher previous body temperature were significant protective factors against core hypothermia. In this analysis independent predictors of hypothermia on admission to ICU were: magnitude of surgery, use of general anesthesia or combined epidural and general anesthesia, total intravenous crystalloids administrated and total packed erythrocytes administrated, anesthesia longer than 3 hours and SAPS II scores. In multiple logistic regression analysis significant predictors of hypothermia on admission to the ICU were magnitude of surgery (OR 3.9, 95% CI, 1.4–10.6, p = 0.008 for major surgery; OR 3.6, 95% CI, 1.5–9.0, p = 0.005 for medium surgery), intravenous administration of crystalloids (in litres) (OR 1.4, 95% CI, 1.1–1.7, p = 0.012) and SAPS score (OR 1.0, 95% CI 1.0–1.7, p = 0.014); higher previous temperature in ward was a significant protective factor (OR 0.3, 95% CI 0.1–0.7, p = 0.003). Hypothermia was neither a risk factor for hospital mortality nor a predictive factor for staying longer in ICU.

**Conclusion:**

The prevalence of patient hypothermia on ICU arrival was high. Hypothermia at time of admission to the ICU was not an independent factor for mortality or for staying longer in ICU.

## Background

Hypothermia is defined as a core body temperature of less than 35°C and may be classified as mild (32°C – 35°C), moderate (28°C – 32°C) and severe (<28°C) [1-4].

Hypothermia promotes impairment and abnormalities in various organs and systems that can lead to a decrease in oxygen release into the tissues [5] and include depression in myocardial contractility, peripheral vasoconstriction [6,7] ventilation-perfusion mismatch, increased blood viscosity and shifts to the left in the oxyhemoglobin-dissociation curve [8,9]. Hypothermia also reduces platelet function [10-12] and decreases the activation of the coagulation cascade [13] and may result in coagulopathy [14,15].

Inadvertent core hypothermia is often found in the immediate postoperative period especially in high risk surgical patients [9,16]. Anesthesia impairs central thermoregulation, allowing re-distribution of body heat [17,18]. Cool ambient temperatures and high volume fluid administration accelerate loss of heat to the environment. The initial response to cold stress is to generate and conserve temperature via the activation of the sympathetic nervous system. In the postoperative period, hypothermia is often complicated by shivering and peripheral vasoconstriction. The consequences of shivering include an increase in cardiac and systemic energy demand, raising oxygen consumption and carbon dioxide production and an increase in cardiac work [19,20]. Randomized control trials have proven that mild hypothermia can lead to an increased incidence of surgical wound infection [21], myocardial ischemia and cardiac arrhythmias [22], impaired coagulation and immune response [11,23]; other complications include enhanced anesthesia drug effects [24,25], shivering [20,26], prolonged recovery room stays and delayed discharge from the post anesthesia care and from hospital [27].

On the basis of review of the literature, the most important risk factors for the development of hypothermia in the perioperative period include age [28,29], sex [9,30,31], body weight and body surface area [16,32], preoperative body temperature [16,33] history of diabetic neuropathy [34], emergency surgery [30], ASA physical status [16], surgical procedure in which major body cavities or major vessels are exposed [9,16], anesthetic technique [31] warming method [35], amount of intravenous replacement [30], duration of anesthesia or surgery [9,16,30,31] and ambient operating room temperature [16,28].

With the progress in surgery and anesthesia more advanced technology was introduced, procedures are using more complex techniques, lasting longer and sometimes are made with larger incisions. These procedures are done in older and severely ill patients who present significant underlying medical problems and have sometimes undergone previous extensive surgery. The choice of anesthesia technique is also changing and combined epidural and general anesthesia are being used more often. These are reasons that may increase risk of intraoperative and immediate postoperative hypothermia. Whereas effective warming measures are available [35] and more information about the adverse effects of hypothermia has been reported, the incidence of hypothermia at the time of admission to the surgical ICU is still frequent [22,29,30]. Re-identifying significant predictive factors would help in decreasing this incidence. Such information could be helpful to prevent unnecessary risks and adverse outcomes and could decrease the current frequent incidence of hypothermia.

The purpose of this study was to estimate the prevalence of hypothermia on admission to a surgical ICU and retrospectively identify their clinical predictors. A second objective was to prospectively evaluate the outcome measured in terms of ICU length of stay (LOS) and hospital mortality.

## Methods

The protocol was approved by our institutional review board, and written consent was obtained from the patients. All consecutive postoperative patients, aged 18 years or older, who underwent scheduled or emergency noncardiac surgery, admitted to a nine bed surgical ICU of a tertiary care hospital during a three month period between April and July 2004, were eligible for the study. Temperature of the operating room was not controlled for every patient because the air conditioning system is automatically settled to a temperature room from 20°C to 22°C. This fact was confirmed by a pilot study in which operating room temperature was measured using thermocouples placed near patients but away from any heat-generating equipment.

Core temperature (Tc) was measured by an infrared tympanic membrane thermometer (ThermosScan^® ^Type 6014 Pro 3000, WelchAllyn, with an accuracy of ± 0.03°C in the range of 20°C–42.2°C – Welch Allyn Medical Products, NY, USA) [36,37]. This instrument was maintained and calibrated in accordance with the manufacturer's guidelines. These tympanic membrane temperatures were obtained before surgery in the ward, on arrival at ICU and every two hours until 6 hours after admission by experienced nurses trained to measure tympanic membrane temperatures.

The following clinical variables were recorded on admission to the ICU: age, sex, body weight and height, preoperative body temperature, ASA physical status, emergency or scheduled surgery, magnitude of surgical procedure as major (surgery in which body cavities or major vessels are exposed to ambient temperature such as major abdominal, thoracic, major vascular, thoracic spine surgery with instrumentation, or hip arthroplasty), medium (surgery in which body cavities are exposed to a lesser degree such as appendectomy), and minor surgery (superficial surgery), anesthesia technique, amount of intravenous crystalloids, colloids, packed erythrocytes and fresh frozen plasma administered during surgery, use of temperature monitoring and warming techniques, and duration of the anesthesia.

The LOS and the mortality in ICU and in hospital were also recorded for all patients, as well as the Simplified Acute Physiology Score II (SAPS II) was calculated [38].

The prevalence of core hypothermia on admission to the ICU and its 95% Confidence Interval (CI) were calculated by using the cut off point of Tc 35.0°C. That was used to classify patients as either hypothermic (Tc<35°C on admission to ICU) or normothermic (Tc ≥ 35°C on admission to ICU). All hypothermic patients were treated with passive and active external rewarming measures that include synthetic covers and a convective air-warming system using heated air blanket units. The two groups were compared to assess the relationship between each clinical predictor and core hypothermia using univariate analysis performed by simple binary logistic regression with an odds ratio (OR) and its 95% CI and independent sample t test, χ^2 ^test or Fisher's exact test. A multiple regression binary logistic with forward conditional elimination was used to examine covariate effects of each factor on core hypothermia, ICU LOS and hospital mortality and to calculate OR and their 95% CI. Covariates with a univariate p < 0.1 in the respective univariate analysis were entered in these models. In the model for ICU LOS and hospital mortality the categorical variable temperature on admission was also entered. A two-sided significance level of 0.05 was used for all analyses.

Quantitative variables are presented as mean ± SD. All analyses were performed using SPSS for Windows (version 12.0, Chicago, IL).

## Results

All 185 patients (Table 1) were included in the study. The mean ( ± SD) admission Tc was 34.7°C ± 1.0°C (range, 32.1°C–38.2°C, first quartile, 34.1°C, third quartile 35.4°C). Prevalence of core hypothermia on ICU admission was 57.8% (95% CI, 54.2%-61.6%). Ten patients were admitted with a Tc <33.0°C.

**Table 1:** Patient baseline characteristics (n = 185)

Variable	mean ± SD or median and range or number (%)
Age	Median 67, mean 66.0 ± 12.6 (range, 25 – 94)
<65 / ≥ 65	79 / 106
Male / Female	112 (60.5) / 73 (39.5)
Body mass index (Kg/m2)	25.4 ± 5.9 (range, 15.6 – 57.6)
Previous temperature in the ward (°C)	36.37 ± 0.49 (range, 35.00 – 38.60)
ASA Physical status	
I	5 (2.7)
II	72 (38.9)
III	90 (48.6)
IV	18 (9.7)
Emergency surgery	29 (15.7)
Magnitude of surgery	
Minor	36 (19.5)
Medium	45 (24.3)
Major	104 (56.2)
General anesthesia	158 (85.4)
Regional anesthesia	19 (10.3)
Combined anesthesia	7 (3.8)
Temperature monitoring	22 (11.9)
Warming technique	81 (43.8)
Intravenous crystalloids (L.)	2.85 ± 1.65 (range 0.20 – 10.50)
Intravenous colloids (L.)	0.09 ± 0.26 (range 0 – 1.500)
Packed erythrocytes (Units)	0.7 ± 1.3 (range, 0 – 7)
Fresh frozen plasma (Units)	0.2 ± 0.9 (range, 0 – 9)
Duration of anesthesia (min.)	218 ± 108 (range, 44 – 660)
> 180 min.	94 (50.8)
Temperature on admission	34.69 ± 1.02 (32.1 – 38.2)
≤ 35	107 (57.8)
Temperature 2 hours after admission	35.34 ± 0.85 (32.4 – 38.3)
≤ 35	48 (25.9)
Temperature 4 hours after admission	35.90 ± 0.80 (32.5 – 38.4)
≤ 35	16 (8.8)
Temperature 6 hours after admission	36.12 ± 0.73 (33.4 – 38.5)
≤ 35	9 (5)
Score of Acute Physiologic system (SAPS II)	24.4 ± 14.0 (range 3–74)
Length of ICU stay (days)	median 0.92; percentile 25, 0.79; percentile 75, 2.11 range (0.08–82)
> 2 days	47 (25.4)
Length of hospital stay (days)	median 15; percentile 25, 10; percentile 75, 29.5 range 1–170
Mortality in the ICU	14 (7.60)
Mortality in the Hospital	29 (15.70)

The prevalence of hypothermia two hours, four hours and six hours after admission was respectively 26,0% (95% CI, 25.6%-26.3%), 8.8% (95% CI, 8.6%-9.1%) and 5,0% (95% CI, 4.8%-5,1%).

According to univariate analysis (Table 2), age, sex, body weight or body mass index, amount of intraoperative colloids and plasma volume, ASA physical status, emergency surgery, surgery longer than 180 minutes were not predictive of core hypothermia at ICU admission.

**Table 2:** Univariate Analysis of categorical and continuous predictors of core hypothermia

**Variable**	**Hypotermic / non-hypothermic (n° or mean ± sd)**	**Odds ratio (95% CI)**	**p-value**
Age (years)			
< 65	44/35	1	
≥ 65	63/43	0.9 (0.5–1.6)	0.611
Gender			
Female	39/34	1	
Male	68/44	0.7 (0.4 – 1.4)	0.327
Body weight (Kg)	69.19 ± 16.79 / 68.49 ± 16.06	1,0 (1.0 – 1.0)	0.771
Body mass index (Kg/m2)	25.30 ± 5.77 / 25.58 ± 6.14	1,0 (0.9 – 1.0)	0.750
Previous temperature in the ward (°C)	36.28 ± 0.44 / 36.49 ± 0.54	0.4 (0.2 – 0.8)	0.007
ASA Physical status			
I	3/2	1	
II	43/29	1.5 (0.2 – 11.2)	0.693
III	52/38	1.5 (0.5 – 4.2)	0.457
IV	9/9	1.4 (0.5 – 3.8)	0.544
Emergency surgery	18/11	0.8 (0.4 – 1.8)	0.616
Magnitude of surgery			
Minor	11/25	1	
Medium	26/19	3.1 (1.2 – 7.8)	0.016
Major	70/34	4.7 (2.1 – 10.6)	< 0.001
General anesthesia	93/65	6,0 (1.9 – 18.8)	0.002
Regional anesthesia	4/15	1	
Combined anesthesia	6/1	22.5 (2.1–244.8)	0.011
Temperature monitoring	19/3	0.2 (0.1–0.7)	0.009
Warming technique	55/26	0.5 (0.3 – 0.9)	0.015
Intravenous crystalloids (L.)	3.21 ± 1.75 / 2.37 ± 1.35	1.4 (1.2 – 1.8)	0.001
Intravenous colloids (L.)	0.08 ± 0.26 / 0.10 ± 0.26	0.7 (0.2 – 2.2)	0.565
Packed erythrocytes (Units)	0.92 ± 1.45 / 0.37 ± 0.81	1.6 (1.1 – 2.2)	0.006
Fresh frozen plasma (Units)	0.27 ± 0.81 / 0.19 ± 1.08	1.1 (0.8 – 1.6)	0.575
Duration of anesthesia (min.)			
≤ 180 min.	46/45	1	
> 180 min.	61/33	1.8 (1.0 – 3.3)	0.049
Score of Acute Physiologic system (SAPS II)	26.45 ± 14.95 / 21.51 ± 12.14	1.0 (1.0 – 1.1)	0.020

Temperature monitoring (OR 0.2, 95% CI 0.1–0.7, p = 0.009), use of warming techniques (OR 0.5, 95% CI 0.3–0.9, p = 0.015) and higher previous body temperature (OR 0.4, 95% CI 0.2–0.8, p = 0.007) were significant protective factors against core hypothermia.

Significant independent predictors of hypothermia on admission were the magnitude of surgery (OR 3.1, 95% CI 1.2–7.8, p = 0.016 for medium surgery; OR 4.7, 95% CI 2.1–10.6, p < 0.001 for major surgery), use of general anesthesia or combined epidural and general anesthesia (OR 6.0, 95% CI, 1.9–18.8, p = 0.002 for general anesthesia; OR 22.5, 95% CI 2.1–244.8, p = 0.011 for combined epidural and general anesthesia), amount of intravenous crystalloids (OR 1.4, 95% CI 1.2–1.8, p = 0.001) and number of total units of packed erythrocytes (OR 1.6, 95% CI 1.1–2.2, p = 0.006), anesthesia longer than 3 hours (OR, 1.8; 95% CI, 1.0–3.3, p= 0.049) and SAPS II scores (OR 1.0, 95% CI 1.0–1.1, p = 0.020).

Table 3 displays the results of the multiple regression binary logistic analysis. In this model higher preoperative body temperature (OR 0.3, 95% CI 0.1–0.7, p = 0.003) is seen as a significant protective factor against core hypothermia. In this analysis the higher the SAPS II of the patient is, the higher the risk of core hypothermia (OR 1.0, 95% CI 1.0–1.7, p = 0.014). The magnitude of surgery (OR 3.9, 95% CI, 1.4–10.6, p = 0.008 for major surgery; OR 3.6, 95% CI, 1.5–9.0, p = 0.005 for medium surgery) and total intravenous crystalloids (in litres) (OR 1.4, 95% CI, 1.1–1.7, p = 0.012) were considerably significant risk factors.

**Table 3:** Predictors of core hypothermia by multiple logistic regression

**Variable**	**Odds ratio (95% CI)**	**p-value**
Magnitude of surgery		
Medium	3.6 (1.5 – 9.0)	0.005
Major	3.9 (1.4 – 10.6)	0.008
Intravenous crystalloids (L)	1.4 (1.1 – 1.7)	0.012
Previous temperature in the ward	0.3 (0.1 – 0.7)	0.003
Score of Acute Physiologic system (SAPS II)	1.0 (1.0 – 1.7)	0.014

Model adjusted to: previous temperature in the ward, magnitude of surgical procedure, type of anesthesia, use of temperature monitoring, use of warming technique, total intravenous crystalloids, total packed erythrocytes, duration of anesthesia and SAPS II score

No significant relationship between hypothermia at ICU admission and LOS in hospital or in ICU was to be found.

As can be seen in table 1, ICU LOS varied from 0.1 to 82 days with median of 0,9 days (percentile 25, 0.8 days and percentile 75, 2.1 days). The percentage of patients who stayed in ICU longer than 2 days were 25.4% (n = 47).

Multiple regression logistic analysis was used to examine covariate effects of each factor on ICU LOS (table 4). In this analysis the regression model included temperature on admission and all variables that showed statistical significance in the univariate analysis made for predictors of hypothermia and for mortality. This analysis showed that significant risk factors for staying longer in ICU were SAPS II (OR 1.1, 95% CI 1.1–1.1, p < 0.001), ASA physical status (OR 3.4, 95% CI 1.3–9.0, p = 0.012 for ASA III/IV patients) and amount of intravenous crystalloids administered (OR 1.3, 95% CI 1.0–1.7, p = 0.023).

**Table 4:** Predictors of ICU LOS longer than two days by multiple logistic regression

**Variable**	**Odds ratio (95% CI)**	**p-value**
ASA III/IV	3.4 (1.3 – 9.0)	0.012
SAPS II	1.1 (1.1 – 1.1)	<0.001
Intravenous crystalloids (L.)	1.3 (1.0–1.7)	0.023

Model adjusted to: temperature on admission, previous temperature in the ward, magnitude of surgery, type of anesthesia, use of temperature monitoring, use of warming technique, total intravenous crystalloids, total packed erythrocytes, duration of anesthesia, BMI, ASA and SAPS II score.

Fourteen (7.6%) patients died in ICU and 29 (15.7%) died during their hospitalization.

According to univariate analysis (table 5), age, sex, anesthesia technique, use of a perioperative warming technique, temperature monitoring, duration of anesthesia or surgery were not independent risk factors for mortality in the hospital, as temperature was not a risk factor on admission, neither at two, four and six hours after arrival at ICU.

**Table 5:** Univariate Analysis of categorical and continuous predictors of mortality in the hospital

**Variable**	**nonsurvivors / survivors n° or mean ± SD**	**Odds ratio (95% CI)**	**p-value**
Age (years)			
< 65	14/65	1	
≥ 65	15/91	1.3 (0.6 – 2.9)	0.510
Gender			
Male	19/93	0.8 (0.3 – 1.8)	0.551
Female	10/63		
Body weight (Kg)	63.2 ± 11.8 / 69.8 ± 16.9	1.0 (0.9 – 1.0)	0.044
Body mass index (Kg/m2)	23.1 ± 3.83 / 25.8 ± 6.1	0.9 (0.8 – 1.0)	0.019
ASA Physical status			
I/II	8/ 69	1	
III/IV	21/87	2.1 (0.8 – 5.0)	0.10
Emergency surgery	13/16	7.1 (2.9 – 17.4)	<0.001
Magnitude of surgery			
Minor	2/34	1	
Medium	11/34	3.1 (0.7 – 14.2)	0.146
Major	16/88	5.5 (1.1 – 26.7)	0.034
Temperature monitoring	5/17	1.7 (0.6 – 5.1)	0.337
Warming technique	10/71	1.6 (0.7 – 3.6)	0.274
Intravenous crystalloids (L.)	2.88 ± 1.59 / 2.85 ± 1.66	1.0 (0.8 – 1.3)	0.904
Intravenous colloids (L.)	0.16 ± 0.33 / 0.08 ± 0.24	2.4 (0.7 – 8.7)	0.174
Packed erythrocytes (Units)	0.83 ± 1.14 / 0.66 ± 1.27	1.1 (0.8 – 1.5)	0.508
Fresh frozen plasma (Units)	0.17 ± 0.54 / 0.25 ± 0.99	0.9 (0.5 – 1.5)	0.683
Duration of anesthesia (min.)	204 ± 112 / 221 ± 107		
≤ 180 min.	16/75	1	
> 180 min.	13/81	1.3 (0.6 – 3.0)	0.484
Temperature on admission			
≤ 35	20/87	1	
> 35	9/69	0.6 (0.2 – 1.3)	0.190
Score of Acute Physiologic system (SAPS II)	41.41 ± 17.86 / 21.20 ± 10.54	1.1 (1.1 – 1.1)	<0.001
Length of ICU stay (days)			
≤ 2 days	10/128	1	
> 2 days	19/28	8.7 (3.7 – 20.7)	<0.001
Length of hospital stay (days)	45.7 ± 41.8 / 21.8 ± 21.9	1.0 (1.0 – 1.0)	<0.001

Statistically significant independent risk factors for hospital mortality were low body weight (OR 1.0, 95% CI 0.9–1.0, p = 0.044) and low body mass index (OR 0.9, 95% CI 0.8–1.0, p = 0.019), emergency surgery (OR 7.1, 95% CI 2.9–17.4, p < 0.001), major surgery (OR 5.5, 95% CI 1.1–26.7, p = 0.034), high SAPS II scores (OR 1.1, 95% CI 1.1–1.1, p < 0.001), longer stay in ICU (OR 8.7, 95% CI 3.7–20.7, p < 0.001 for ICU LOS longer than 2 days) and in the hospital (OR 1.0, 95% CI 1.0–1.0, p < 0.001).

The multiple regression logistic analysis (table 6) showed that considerably significant factors predicting death in the hospital were higher SAPS scores (OR 1.1, 95% CI 1.0–1.1, p < 0.001) and ICU LOS (OR 1.2, 95% CI 1.1–1.3, p = 0.003). This analysis showed that these were the factors that more significantly predicted death in the in-hospital setting.

**Table 6:** Predictors of mortality by multiple logistic regression

**Variable**	**Odds ratio (95% CI)**	**p-value**
SAPS II	1.1 (1.0 – 1.1)	<0.001
Length of ICU stay (days)	1.2 (1.1 – 1.3)	0.003

Model adjusted to: temperature on admission to the ICU, BMI, body weight, magnitude of surgery, type of surgery, SAPS II score, ICU LOS and hospital LOS.

## Discussion

Only noncardiac surgical patients were enrolled in the study. Since none of them underwent a neurosurgical procedure, hypothermia as a therapeutic tool had not been applied.

The prevalence of hypothermia on arrival at ICU was important and proved higher than in other studies [6,9,16,29,30] although not all of them have chosen the same defining criteria to consider patients hypothermic.

In this study, warmer preoperative body temperature is considered a significant protective factor for hypothermia what was also demonstrated previously [16,33]. This result supports the suggestion that efforts should be done to increase body temperature before surgery. In this context is the idea of increasing operating room temperature to prevent heat loss. Various studies have showed that increasing operating room temperature could prevent core hypothermia [16,39], however, when the operating room is too warm, that is not a comfortable working environment and may even increase the risk of infection [21,40].

Previous studies have shown that older patients [9,30,31] and those with higher ASA physical status [16] had an increased risk for hypothermia. Our study could not confirm these results. We think that may be explained because these patients had more often benefited from any perioperative method of warming and monitoring. In fact, monitoring temperature and the use of techniques to warm patients have demonstrated as protective measures according to our results.

We found that as measured by SAPS II score the more severely ill the patients, the more the probability of arriving hypothermic at ICU.

Although the objective of this study was exploring preadmission clinical factors to hypothermia, we included SAPS in the list of predictive values because this way we could have a measure of severity of illness in the patients admitted to ICU. Measured after admission to ICU, this score reflects not only the pre admission physiologic alterations but also variables like age and co morbidities that were not altered with admission. Only with this premises should the predictive value of SAPS II be evaluated.

Hypothermia is a common occurrence of postoperative period particularly if major body cavities are exposed for long periods of time and other important contributors like the use of intraoperative large amount of intravenous fluids, prolonged operative time and use of general anesthesia or combined anesthesia (general and loco regional anesthesia) are present. In our study, the magnitude of surgery was an independent risk factor for core hypothermia what is in agreement with other studies [9,16] and like other studies [9,16,30,31] that found that duration of anesthesia or surgery are risk factors for hypothermia, we concluded that anesthesia lasting longer than 3 hours was a predictor of core hypothermia with statistical significance. If patients undergoing major procedures with long lasting anesthesia became hypothermic more often, they were probably not warmed actively as we could suspect from the active warming rate and rate of temperature monitoring of this set of patients.

We concluded that the amount of intravenous intraoperative crystalloids was a significant risk factor for developing hypothermia on arrival on ICU and that was already identified in previous studies [9,30]. In fact the infusion of crystalloid solutions at room temperature may significantly contribute to intraoperative hypothermia because warming fluids to core temperature requires body heat. Several studies had demonstrated that infusion of warmed fluids helps in the prevention of hypothermia and reduces the incidence of postoperative shivering [41,42]. A limitation of our protocol was the absence of data about how often had been used warmed intraoperative fluids and what methods were used to warm them.

We found that general anesthesia added significant intraoperative risks for core hypothermia which is in accordance with previous reports showing a frequent incidence of hypothermia with general anesthesia [43,44]. Our results showed a closer relationship concerning the incidence of core hypothermia in the presence of combined epidural anesthesia and general anesthesia and those patients in whom this technique was used were significantly hypothermic at the admission to ICU.

Inadvertent hypothermia during anesthesia is by far the most common perioperative thermal disturbance; this results from a combination of impaired thermoregulation and exposure to the cold environment of the operating room [5]. Prevention of hypothermia reduces anesthesia-related morbidity; so body temperature should be carefully monitored, regardless of anesthetic technique [45] and active warming should be used more frequently because it is a simple and effective technique to avoid hypothermia. In our hospital, and as we could demonstrate with this study, warming rates were very low and the rate of temperature monitoring was even lower, predisposing patients to hypothermia. Like Sessler stated "the minor and major complications of hypothermia are thus well documented. In some patients mild hypothermia is likely to be dangerous. In others it will be uncomfortable and slow recovery". Like him we propose that intraoperative core hypothermia should be avoided and we think the proposed management guidelines should always be observed [46].

Unlike found in an older study [29], we could not show any relationship between hypothermia on arrival at ICU and longer staying in ICU.

We could confirm the results of others about severity of illness of patients as measured by ASA physical status and SAPS II as predictors of prolonged stays in ICU [47,48] and the same happens with the greater amount of intraoperative intravenous fluid administration [16].

Overall mortality in our study was 7,6% in ICU and 15,7% in hospital, values that are within the range expected from the corresponding standard mortality ratios of SAPS II [38,49]. Hypothermia on admission to ICU was not an independent factor for mortality.

Body mass index, ASA physical status, type of surgery, major surgery, SAPS II score and ICU and hospital LOS were found as statistically significant clinical predictors of death, according to the univariate analysis; whereas according to multiple logistic regression analysis, only the severity of illness of patients as measured by SAPS II and longer ICU stay were confirmed as significantly predictors of death.

A limitation of our protocol is that core temperatures were estimated from the aural canal using an infrared thermometer. Infrared measurements may introduce a degree of variability that could be avoided with carefully positioned thermocouples [37]. However, previous observations state that infrared thermometers are very accurate for determining a patient's temperature when used by those who routinely perform thermometry in hospitalized patients and they can provide accurate estimative of core body temperature [50,51].

A controversial point of our study is the considered definition of hypothermia as Tc < 35°C. Others have chosen the same value of core temperature or even a lower one as definition of hypothermia [3,6,9]. Meanwhile many approaches have used 36°C as the cut off point between hypothermia and normothermia in ICU [52] and studies have considered it in their methodology [16,53].

Improved awareness about perioperative hypothermia and its complications with a strictly adherence to the recommendations about temperature monitoring and thermal management guidelines [46] should play a role in decreasing adverse outcome. It may be advantageous to take steps in the way to decrease prevalence of hypothermia and conduct a study to validate this hypothesis.

## Conclusion

The prevalence of patient hypothermia on arrival at intensive care is very high but, in our results, is not an independent factor for mortality or for staying longer in ICU. The magnitude of surgery, the severity of disease, longer anesthesia periods, the technique of combined epidural and general anesthesia, large amounts of fluids in the operative setting, not using temperature monitoring and a technique to warm patients tend to be risk factors for hypothermia.

All effort to prevent hypothermia should be done, including the more frequently use of body temperature monitoring and methods to warm patients.

## Competing interests

None of the authors have any financial or other relationship that might influence the objectivity when performing the study or preparing the manuscript.

## Authors' contributions

FA participated in conception, design, acquisition of the data, analysis of the data, statistical analysis, critical revision of the manuscript and supervision.

MC participated in conception, design, acquisition of the data, analysis of the data and critical revision of the manuscript.

CS participated in analysis of the data, statistical analyses and drafting of the manuscript.

AN and NL participated in acquisition of the data, analysis of the data and revision of the manuscript.

## Pre-publication history

The pre-publication history for this paper can be accessed here:


